# Genomic Biomarker Heterogeneities between SARS-CoV-2 and COVID-19

**DOI:** 10.3390/vaccines10101657

**Published:** 2022-10-02

**Authors:** Zhengjun Zhang

**Affiliations:** Department of Statistics, School of Computer, Data & Information Sciences, University of Wisconsin, Madison, WI 53706, USA; zjz@stat.wisc.edu

**Keywords:** COVID-19 detection, gene-gene interaction, functional effects, competing risks, computational medicine

## Abstract

Genes functionally associated with SARS-CoV-2 infection and genes functionally related to the COVID-19 disease can be different, whose distinction will become the first essential step for successfully fighting against the COVID-19 pandemic. Unfortunately, this first step has not been completed in all biological and medical research. Using a newly developed max-competing logistic classifier, two genes, ATP6V1B2 and IFI27, stand out to be critical in the transcriptional response to SARS-CoV-2 infection with differential expressions derived from NP/OP swab PCR. This finding is evidenced by combining these two genes with another gene in predicting disease status to achieve better-indicating accuracy than existing classifiers with the same number of genes. In addition, combining these two genes with three other genes to form a five-gene classifier outperforms existing classifiers with ten or more genes. These two genes can be critical in fighting against the COVID-19 pandemic as a new focus and direction with their exceptional predicting accuracy. Comparing the functional effects of these genes with a five-gene classifier with 100% accuracy identified and tested from blood samples in our earlier work, the genes and their transcriptional response and functional effects on SARS-CoV-2 infection, and the genes and their functional signature patterns on COVID-19 antibodies, are significantly different. We will use a total of fourteen cohort studies (including breakthrough infections and omicron variants) with 1481 samples to justify our results. Such significant findings can help explore the causal and pathological links between SARS-CoV-2 infection and the COVID-19 disease, and fight against the disease with more targeted genes, vaccines, antiviral drugs, and therapies.

## 1. Introduction

The fluctuations in infection rates of the COVID-19 pandemic have varied from time to time. In the meantime, variants of SARS-CoV-2 have emerged and put scientists and medical practitioners on high alert all the time, and many problems have remained unanswered [1,2,3,4,5,6,7,8,9,10,11]. In addition, there have been new concerns surrounding the COVID-19 disease, e.g., SARS-CoV-2 entering the brain [12], COVID-19 vaccines complicating mammograms [13], memory loss, and ‘brain fog’ [14], amongst others. However, these new concerns are observational and experimental laboratory outcomes, and the genetic bases of those phenomena have not been properly assessed due to a lack of adequate analytical methods to link COVID-19 to the situations. Regarding samples assessed by gene expression profiling, the literature did not point out the significant difference between samples with differential expressions derived from nasopharyngeal (NP) and oropharyngeal (OP) PCR swabs and samples derived from whole blood, as the majority of research work focused on individual genes’ expression levels, especially high expression values. Zhang [15] first applied an innovative algorithm to analyze 126 whole blood samples from COVID-19-positive and COVID-19-negative patients, and reported five critical genes and their competing classifiers, leading to 100% accuracy in classifying all 126 hospitalized patients, including ICU patients, to their respective groups [16]. Zhang [17] further developed a mathematical and biological equivalence between COVID-19 and five critical genes, and proved the existence of at least three transcriptomic data signature patterns and at least seven subtypes. This paper studies gene expression data drawn from NP/OP swab PCR-tested samples with COVID-19 positives and negatives. Surprisingly, we found that the functional effects of those five critical genes, ABCB6, KIAA1614, MND1, SMG1, and RIPK3, found by Zhang [15,17], no longer play a decisive role in NP/OP swab PCR samples. At first glance, this observation seems unhelpful, or even casts doubt on the study’s methodology, genomics, and epigenetics. However, careful thought confirms that this observation perfectly suggests the relationship between whole blood samples and NP/OP swab PCR samples. The former (whole blood) stands for the essence of the disease, while the latter (NP/OP) stands for the point of the phenomenon. Metaphorically, let us consider water quality and mineral examination with samples from the deep sea and samples from the shoreside. The samples from the deep sea represent the meta contents and functions of the sea, while the samples from the shoreside likely contain polluted objects from the sea bank. Additionally, the structures of the deep sea will have changed along the waves. As a result, samples from the deep sea and samples from the shoreside will provide very different information, with an exception in the case of the whole sea being evenly cleaned or polluted. Here, deep-sea samples correspond to whole blood samples, while shoreside samples correspond to NP/OP swab PCR samples, which explains the significant difference inferred from the studies by Zhang [15,17] and this study.

On the other hand, our new finding calls forth an old question: whether to treat the symptoms, cure the root cause, or both. Zhang [17] argues that the existence of a genomic signature pattern has to be solved to end the disease, i.e., it is about curing the root cause. On the other hand, this paper is about treating the symptoms. These two types of research reinforce each other, and both are important to current studies of diseases (any types).

The studies [15,17,18,19,20,21] applied an innovative algorithm to study classifications of COVID-19 patients, breast cancer patients, lung cancer patients, colorectal cancer patients, and liver cancer patients, and gained the highest accuracy (nearly 100%) among eleven different study cohorts with thousands of patients. The high accuracy establishes a mathematical and biological equivalence between the formed classifiers and the disease, which shows that the study method was effective, informative, and robust. These applications are advanced as they lead to new, interpretable, and insightful functional effects of genes linked to the diseases. Using the breast cancer study [18] as an example, it was found that the known eight famous genes—BRCA1, BRCA2, PALB2, BARD1, RAD51C, RAD51D, and ATM—in breast cancer research and practice actually lead to very low accuracy in predicting breast cancer status at the stage of diagnosis. Table 6 in the paper [18] demonstrated that any of these eight genes are very weakly correlated, at most 0.341, with the high-performance biomarkers/genes identified in the study [18]. The findings using our new innovative approach (max logistic classifier) could be the key factor in achieving breakthroughs against diseases. Due to the limitation of the existing analysis methods and the limited knowledge of the diseases, the fundamental functional effects of genes associated with the disease could not be discovered even though the truth in the collected data has existed for a long time, and the chances of discovering the truth have been wasted. Conducting new experiments, producing new data, and applying the same analysis methods are simply repeating, making the same errors of finding suboptimal (even sometimes misleading) answers. For example, it has been reported by the C.D.C. that vaccine effectiveness against medically attended outpatient ARIs associated with the influenza A (H3N2) virus was 16% [22]. Though the efficacy of a vaccine involves many factors, e.g., the rate of virus mutation, recombination, or aspects of its biological cycle, other than by technical aspects of classification or design studies, identifying fundamental genomic/genetic gene–gene interactions can be intrinsic. This paper uses the innovative method of studying differential expressions of human upper respiratory tract gene expressions from 93 COVID-19-positive patients and 141 patients with other acute respiratory illnesses, with or without viral infections [23], and to study host gene expression among RNA-sequencing profiles of nasopharyngeal swabs from 430 individuals with SARS-CoV-2 infection and 54 negative controls [24]. In addition to these two datasets, we will study an additional twelve datasets, including blood-sampled datasets and Omicron variants. The details, including how to perform cross-validation with heterogeneous datasets that have not been studied, will be discussed in Section 3. Using the first dataset, we identify two genes, ATP6V1B2 and IFI27, critical in the transcriptional response to SARS-CoV-2 infection. The gene IFI27 was also identified by Mick et al. (2020) [23] but did not enter their final classifiers. In the analysis of the first dataset, a combination of these two genes with RIPK3 [15] can lead to an overall accuracy of 87.2%, a sensitivity of 76.3%, and a specificity of 94.3%, and a combination of these two genes with one of the further three genes BTN3A1, SERTAD4, and EPSTI1 can lead to an overall accuracy of 89.74%, the sensitivities ranged between 89.25~93.55%, and the specificities between 87.24~90.12%, which are higher than the classifiers in the literature. Using these two genes and one other gene together can easily achieve an overall accuracy between 87.2% and 89.74%, revealing that these two genes can be fundamental. Combining all these five genes can achieve an overall accuracy of 91.88%, a sensitivity of 94.62%, and a specificity of 90.08%, higher than the classifiers with 10 genes or more in the literature. Many other combinations will be illustrated in the Data Section. These performance results from different combinations indicate that COVID-19 can have many different variants. Unlike the studies by Zhang [15,17], the accuracy from any combinations applied to NP/OP swab PCR gene expressions has not reached up to 100%. There are three possible reasons, e.g., (1) the samples themselves were false positives or false negatives from NP/OP swab PCR tests; (2) sample signals were weak, and counts were inaccurate; or (3) experimental conditions varied. Nevertheless, given the superior performance in the first dataset, the findings shed light on studying SARS-CoV-2 and infections.

These two critical genes, ATP6V1B2 (ATPase H+ Transporting V1 Subunit B2) and IFI27 (Interferon Alpha Inducible Protein 27), had previously been reported to be associated with several diseases. For example, de novo mutation in ATP6V1B2 was found to impair lysosome acidification and cause dominant deafness-onychodystrophy syndrome, while IFI27 was found to discriminate between influenza and bacteria in patients with suspected respiratory infection [25], among others. In addition, a recent study found that SARS-CoV-2 appeared to persist in organs throughout the body for months [26].

The significant differences in gene functional effects, gene–gene interactions, and gene-variant interactions between whole-blood-sampled gene expressions and NP/OP swab PCR-sampled gene expressions reveal that ATP6V1B2 and IFI27 are associated with SARS-CoV-2, which points to a new optimal direction of developing more effective vaccines and antiviral drugs. On the other hand, the functional effects of ABCB6, KIAA1614, MND1, SMG1, and RIPK3 can be critical to understanding the disease.

The contributions of this paper include: (1) signifying the genomic difference between NP/OP swab PCR samples and whole blood samples (hospitalized patients); (2) identifying single-digit critical genes (ATP6V1B2, IFI27, BTN3A1, SERTAD4, and EPSTI1), which are a transcriptional response to SARS-CoV-2; (3) presenting interpretable functional effects of gene–gene interactions and gene–variant interactions using explicitly mathematical expressions; (4) presenting graphical tools for medical practitioners to understand the genomic signature patterns of the virus; (5) making suggestions on developing more efficient vaccines and antiviral drugs; (6) identifying potential genetic clues to other diseases due to COVID-19 infection. The remainder of the paper is organized as follows. First, Section 2 briefly reviews the studying methodology. Next, Section 3 reports the data source, analysis results, and interpretations. Section 4 offers insights of an additional twelve COVID-19 studies. Finally, Section 5 concludes the study.

## 2. Methodology

Many types of medical research, especially gene expression data-related, apply classical logistic regression as a starting base, then combine this with implementations of advanced machine learning methods. However, Teng and Zhang (2021) [27] point out that classical logistic regression can only model absolute treatments, not relative treatments. As a result, it has led (and will lead) to many supposedly efficient trials being wrongly concluded as inefficient. Four clinical trials, including one COVID-19 study trial, were illustrated in their paper. Their new AbRelaTEs regression model for medical data is much more advanced than classical logistic regression, as it greatly enhances interpretability and truly personalized medicine computability. Our new study in this paper differs from AbRelaTEs as we do not deal with treatment and control, and we use a new innovative method to study the existence of functional effects of genes associated with SARS-CoV-2.

The competing risk factor classifier has been successfully applied in the literature [15,18,19,20,21]. This section briefly introduces the necessary notations and formulas for self-containing due to the different data structures used in this work. For continuous responses, the literature [28,29,30] deals with max-linear competing factor models and max-linear regressions with penalization. The max-logistic classifier has some connections to the logistic polytomous models but with different structures [31,32,33]. This new innovative approach can be classified as either an AI algorithm or a machine learning algorithm. However, our new approach has an explicit formula and is interpretable.

Suppose $Y_{i}$ is the ith individual patient’s COVID-19 status ($Y_{i}=0$ for COVID-19-free, $Y_{i}=1$ for infected) and $X_{i}^{\left(k\right)}=\left(X_{i1}^{\left(k\right)},X_{i2}^{\left(k\right)},\ldots ,X_{ip}^{\left(k\right)}\right),k=1,\ldots ,K$ are the gene expression values, with p between 15,979 and 35,784 genes in this study. Here, k stands for the kth type of gene expression levels drawn based on K different biological sampling methodologies. Note that most published works set K=1, and hence the superscript (k) can be dropped from the predictors. In this research paper, K=4, as we have two datasets analyzed in Section 3, and in the first dataset, there are other ARIs patients with other viral infections or non-viral infections. Using a logit link (or any monotone link functions), we can model the risk probability $p_{i}^{\left(k\right)}$ of the ith person’s infection status as:

(1) $$\log\left(\frac{p_{i}^{\left(k\right)}}{1-p_{i}^{\left(k\right)}}\right)=\beta_{0}^{\left(k\right)}+X_{i}^{\left(k\right)}\beta^{\left(k\right)}$$

or alternatively, we write

$$p_{i}^{\left(k\right)}=\frac{\exp(\beta_{0}^{\left(k\right)}+X_{i}^{\left(k\right)}\beta^{\left(k\right)})}{1+\exp(\beta_{0}^{\left(k\right)}+X_{i}^{\left(k\right)}\beta^{\left(k\right)})}$$

where $\beta_{0}^{\left(k\right)}$ is an intercept, $X_{i}^{\left(k\right)}$ is a 1×p observed vector, and *β*^(*k*)^ is a p×1 coefficient vector which characterizes the contribution of each predictor (gene, in this study) to the risk.

Considering that there have been several variants of SARS-CoV-2 and multiple symptoms (subtypes) of COVID-19 diseases, it is natural to assume that the genomic structures of all subtypes can be different. Suppose that all subtypes of SARS-CoV-2 may be related to G groups of genes:

(2) $$\Phi_{ij}^{\left(k\right)}=\left(X_{i,j_{1}}^{\left(k\right)},X_{i,j_{2}}^{\left(k\right)},\ldots ,X_{i,j_{g_{j}}}^{\left(k\right)}\right),j=1,\ldots ,G,g_{j}\geq 0,k=1,\ldots ,K$$

where i is the ith individual in the sample, and $g_{j}$ is the number of genes in jth group.

The competing (risk) factor classifier is defined as:

(3) $$\log\left(\frac{p_{i}^{\left(k\right)}}{1-p_{i}^{\left(k\right)}}\right)=max\left(\beta_{01}^{\left(k\right)}+\Phi_{i1}^{\left(k\right)}\beta_{1}^{\left(k\right)},\beta_{02}^{\left(k\right)}+\Phi_{i2}^{\left(k\right)}\beta_{2}^{\left(k\right)},\ldots ,\beta_{0G}^{\left(k\right)}+\Phi_{iG}^{\left(k\right)}\beta_{G}^{\left(k\right)}\right)$$

where $\beta_{0j}^{\left(k\right)}$s are intercepts, $\Phi_{ij}^{\left(k\right)}$ is a $1\times g_{j}$ observed vector, and $\beta_{j}^{\left(k\right)}$ is a $g_{j}\times 1$ coefficient vector which characterizes the contribution of each predictor in the jth group to the risk.

**Remark** **1.** *In (3),* $p_{i}^{\left(k\right)}$ *is mainly related to the largest component* $\beta_{0j}^{\left(k\right)}+\Phi_{ij}^{\left(k\right)}\beta_{j}^{\left(k\right)},j=1,\ldots ,G$ *, i.e., all components compete to take the most significant effect.*

**Remark** **2.** *Taking* $\beta_{0j}^{\left(k\right)}=-\infty ,j=2,\ldots ,G$ *, (3) is reduced to the classical logistic regression, i.e., the classical logistic regression is a special case of the new classifier. Compared with black-box machine learning methods (e.g., random forest, deep learning (convolutional) neural networks (DNN, CNN)) and regression tree methods, each competing risk factor in (3) forms a clear, explicit, and interpretable signature with the selected genes. The number of factors corresponds to the number of signatures, i.e.,* G *. This model can be a bridge between linear models and more advanced machine learning methods (black box) models. However, (3) retains the desired properties of interpretability, computability, predictability, and stability. Note that this remark is similar to Remark 1 in Zhang (2021) [19].*

We have to choose a threshold probability value to decide a patient’s class label in practice. Following the general trend in the literature, we set the threshold to be 0.5. As such, if $p_{i}^{\left(k\right)}\leq 0.5$, the ith individual is classified as being disease-free; otherwise, the individual is classified as having the disease.

With the above-established notations and the idea of a quotient correlation coefficient [34], Zhang (2021) [19] introduced a new machine learning classifier, smallest subset and smallest number of signatures (S4), for K=1. We extended the S4 classifier from K=1 to K=4 as follows:

(4) $$\begin{array}{c} \left(\hat{\beta },\hat{S},\hat{G}\right)={\mathrm{argmin}}_{\beta ,S_{j}\subset S,j=1,2,\ldots ,G}\{{\left(1+\lambda_{1}+\left|S_{u}\right|\right)}^{\overset{K}{\underset{k=1}{\sum }}\overset{n}{\underset{i=1}{\sum }}(I\left(p_{i}^{\left(k\right)}\leq 0.5\right)I\left(Y_{i}=1\right)+I(p_{i}^{\left(k\right)}>0.5)I\left(Y_{i}=0\right))}\\ +\lambda_{2}\left(\left|S_{u}\right|-\frac{\left|S_{u}\right|+G-1}{\left(\left|S_{u}\right|+1\right)\times G-1}\right)\} \end{array}$$

where I(.) is an indicative function, $p_{i}^{\left(k\right)}$ is defined in Equation (3), S={1,2,…,15,979 or 35,784} is the index set of all genes, $S_{j}=\left\{j_{j1},\ldots ,j_{j,g_{j}}\right\}$, j=1,…,G are index sets corresponding to (2), $S_{u}$ is the union of $\left\{S_{j},j=1,\ldots ,G\right\}$, $\left|S_{u}\right|$ is the number of elements in $S_{u}$, $\lambda_{1}\geq 0$ and $\lambda_{2}\geq 0$ are penalty parameters, and $\hat{S}=\left\{j_{j1},\ldots ,j_{j,g_{j}},j=1,\ldots ,\hat{G}\right\}$ and $\hat{G}$ are the final gene set selected in the final classifiers and the number of final signatures.

**Remark** **3.** *The case of *K=1*corresponds to the classifier introduced in Zhang (2021) [19]. The case of*K=1*and*$\lambda_{2}=0$*corresponds to the classifier introduced in Zhang (2021) [15]*.

## 3. Data Descriptions, Results and Interpretations

### 3.1. The Data

The two COVID-19 datasets to be analyzed in this section are publicly available at https://github.com/czbiohub/covid19-transcriptomics-pathogenesis-diagnostics-results (accessed on 26 December 2021) [23] and as GSE152075 [24]. The first dataset contains 15,979 genes, 93 patients with NP/OP PCR swabs who tested positive for COVID-19, 41 patients with viral acute respiratory illnesses (ARIs) and who were COVID-19 negative, and 100 with non-viral acute respiratory illnesses (ARIs) who were COVID-19 negative. The second dataset contains 35,784 genes, 430 individuals with NP/OP PCR swabs with confirmed SARS-CoV-2 infection, and 54 negative controls. We note that many gene expression values in the second dataset are zero.

### 3.2. The Competing Factor Classifiers and Their Resulting Risk Probabilities

Solving the optimization problem (4) among all genes (15,979 and 35,784), various competing classifiers can be identified with different combinations. As discussed in the introduction, the gene expression data used in this study were drawn from NP/OP swab PCR samples (not whole blood samples). Due to likely false positive and negative samples, 100% accurate classifiers with a single-digit number of genes do not exist. Additionally, with the same accuracy (smaller than 100%), different combinations of genes can be candidate classifiers. Therefore, we report the best-performed classifiers in this subsection. After an extensive Monte Carlo search of the best combinations of genes, five genes, ATP6V1B2, IFI27, BTN3A1 (Butyrophilin Subfamily 3 Member A1), SERTAD4 (SERTA Domain Containing 4), and EPSTI1 (Epithelial Stromal Interaction 1), were found to form the S4 classifiers in Equation (4).

Given that the first dataset has three categories (COVID-19 positive, ARIs with non-SARS-CoV-2 viral infection, ARIs without viral infection), we also studied the classification between COVID-19 positives and ARIs with non-SARS-CoV-2 viral infection, and between COVID-19 positives and ARIs without viral infection, which leads to *K*=4 as stated in the prior subsection.

Note that in (3) each individual component itself is a classifier, which has the following form:

(5) $$\beta_{0}+\beta_{1}\times \mathrm{ATP}6V1B2+\beta_{2}\times \mathrm{IFI}27+\beta_{3}\times \mathrm{BTN}3A1+\beta_{4}\times \mathrm{SERTAD}4+\beta_{5}\times \mathrm{EPSTI}1$$

where $\left(\beta_{0},\beta_{1},\ldots ,\beta_{5}\right)$ are coefficients. In the subsequent subsections, we use tables to present individual (${\mathrm{CF}}_{i,j}$) and combined (${\mathrm{CFmax}}_{j}$) classifiers representing (5), where i is the index for a classifier, and j is for a dataset.

The risk probabilities of each component classifier are:

(6) $$P_{i,j}=\frac{\exp\left({\mathrm{CF}}_{i,j}\right)}{1+\exp\left({\mathrm{CF}}_{i,j}\right)}$$

and the risk probabilities based on all *G* component classifiers together are:

(7) $${\mathrm{Pmax}}_{j}=\frac{\exp\left({\mathrm{CFmax}}_{j}\right)}{1+\exp\left({\mathrm{CFmax}}_{j}\right)}$$

### 3.3. First Dataset: Three-Gene Classifiers (G = 1)

Note that the results in this subsection are not from our final best-performed classifiers. We found that a combination of ATP6V1B2 and IFI27 with many other genes can lead to high-accuracy classifiers. We present their performance combined with the remaining genes of this paper’s best subset of five genes and one of the five critical genes found by Zhang [15]. Table 1 and Table 2 summarize the results.

Table 1 and Table 2 show that the coefficient signs of ATP6V1B2 and IFI27 are the same across all individual classifiers, which is a strong indication that they are truly associated with the virus. Although gene RIPK3 plays a key role in the perfect classifier identified in Zhang [15], its performance was inferior to the other three genes identified from NP/OP PCR swab samples in this paper. This phenomenon reflects the discussions in the Introduction that RIPK3 is related to the natural essence of COVID-19, while ATP6V1B2, IFI27, BTN3A1, SERTAD4, and EPSTI1 contain more information about SARS-CoV-2.

We note that BTN3A1 combinations with ATP6V1B2 and IFI27 can have numerous types, which also lead to the same level of accuracy; for SERTAD4, there are numerous combinations with ATP6V1B2 and IFI27, and the same is true for EPSTI1. The coefficients listed in Table 1 are just a particular type of coefficient. Additionally, for EPSTI1, we can achieve different sensitivities and specificities while maintaining the same accuracy. Among four genes (BTN3A1, SERTAD4, EPSTI1, and RIPK3), EPSTI1 performs best in Table 1 and Table 2. This empirical evidence proves that ATP6V1B2 and IFI27 are at the center of the genes associated with SARS-CoV-2.

### 3.4. First Dataset: Five-Gene Classifiers and the Existence of Variants

Our extensive Monte Carlo search lead to the best solution, with an accuracy of 91.82%, to the optimization problem (4) by five genes, i.e., ATP6V1B2, IFI27, BTN3A1, SERTAD4, and EPSTI1, though the solution is not unique. After comparing solutions for all three categories in the first dataset, these five genes stand out. Table 3, Table 4 and Table 5 summarize the results.

In Section 3.3, we forced ATP6V1B2 and IFI27 to be members in each classifier, while the best performance classifiers in this section revealed that they can function separately, which tells us that a gene’s function heavily depends on other genes’ function, i.e., gene–gene interactions, and gene–disease subtype interactions. Furthermore, such a phenomenon suggests SARS-CoV-2 variants/subtypes are heterogeneous. As a result, models without differentiating gene–gene interactions and gene–variant interactions can be suboptimal.

Table 6 demonstrates part of patients’ expression values of the five critical genes, competing classifier factors, and predicted probabilities. Note that due to relatively very large scales in Columns CF1, CF2, and CFmax, they were rescaled by a division of 100 when computing the risk probabilities, as very large values can result in an overflow in computation. The validity of rescaling was justified in Zhang [17].

**Figure 1:**
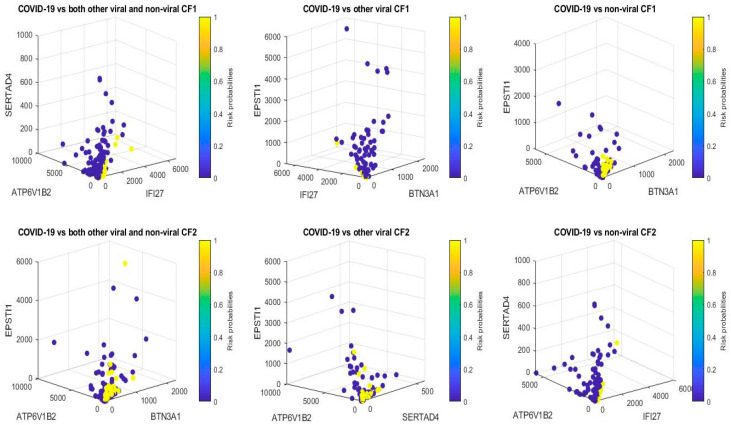
COVID-19 classifiers in Table 3, Table 4 and Table 5: Visualization of gene–gene relationship and gene-risk probabilities. Note that 0.5 is the probability threshold.

Figure 1 presents critical gene expression levels and risk probabilities corresponding to different combinations in the first dataset and Table 3, Table 4 and Table 5. It can be seen that each plot shows the genomic signature pattern and functional effects of the genes involved.

From Table 1, Table 2, Table 3, Table 4 and Table 5, we can immediately see that the coefficient signs associated with ATP6V1B2 are uniformly negative, which shows that increasing the expression level of ATP6V1B2 will decrease the virus (SARS-CoV-2) strength; the coefficient signs associated with IFI27 are uniformly positive, which shows that decreasing the expression level of IFI27 will decrease the virus (SARS-CoV-2) infection strength. Such functional effects of ATP6V1B2 and IFI27 can also be clearly seen in Figure 1 around origins which show that the higher the IFI27 level, the higher the risk probability (yellow color), and the higher the ATP6V1B2 level, the lower the risk probability (blue color). These observations show that ATP6V1B2 and IFI27 are in the circle of genes associated with SARS-CoV-2. BTN3A1 appears three times in Table 3, Table 4 and Table 5 with positive coefficients, which shows that decreasing the expression level of BTN3A1 will decrease the virus (SARS-CoV-2) infection strength. The coefficient signs of SERTAD4 and the coefficient signs of EPSTI1 show both positive and negative values in Table 3, Table 4 and Table 5 depending on how the genes are combined. These phenomena explain the reason SARS-CoV-2 variants have emerged, as variants can be related to different coefficient signs corresponding to genes.

**Figure 2:**
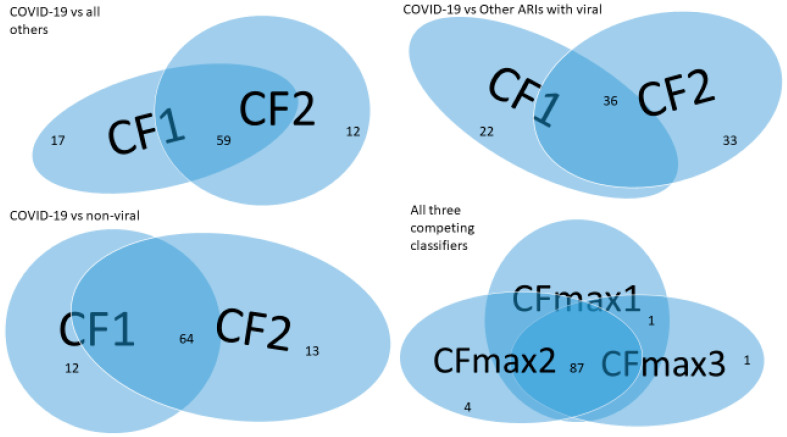
Venn diagram of variants of SARS-CoV-2 (the first dataset): **Top-left** panel is for COVID-19 vs. all others; **Top-right** panel is for COVID-19 vs. other viral infections; **Bottom-left** panel is for COVID-19 vs. non-viral infections; **Bottom-right** panel is for all three together.

Figure 2 is a Venn diagram illustrating each classifier’s performance and the combined classifier. In the Venn diagram, those patients who fall in the intersections are relatively easy to be tested and confirmed positive, while for those who only fall in one category, it is relatively hard to test and confirm their status. Two individual classifiers can be explained as having two COVID-19 tests using two different testing procedures (kits), and with both tests being positive, the probability of infection will be higher depending on the sensitivity and the specificity of each test. Summarizing Table 3, Table 4 and Table 5 and Figure 2, mathematically speaking, SARS-CoV-2 can have 3×3×3×4=108 variants, with some of them being insignificant from the dominant variants and some of them being dominant and having emerged (or will emerge), where the multiplier 3 corresponds to 3 classes in one Venn diagram, and, similarly, other numbers are interpreted. Such an amount of variants may offer a genomic clue to what has been found in Chertow et al. (2021) [26]. We note that the joint functional effects of genes are not directly observable, and the meaning of variants is defined by their joint functional effects. As a result, the variants of the virus are not directly referred to as what has been known in the literature and practice.

Comparing the individual classifiers and combined classifiers among COVID-19 vs. all other infections, COVID-19 vs. ARIs with other viral infections, and COVID-19 vs. ARIs without viral infections, we see that the combined classifier for the case of COVID-19 vs. without viral infections worked the best. We found that some ARIs with other viral infections may be COVID-19 patients, but this was not yet confirmed. Applying the classifier in the bottom-right panel of Figure 2 can achieve a sensitivity of up to 98.94% with a slight loss of specificity.

The five genes, ATP6V1B2, IFI27, BTN3A1, SERTAD4, and EPSTI1, performed better in classifying patients in their respective groups in the first dataset. Therefore, a natural question will be whether or not the accuracies were overestimated. Next, we address this question in two aspects.

In the literature, in order to avoid overfitting data, cross-validation (CV) has been widely utilized in model building and inference. However, this methodology only works when samples are drawn from a homogeneous population. When samples are from heterogeneous populations, CV methods will lead to inaccurate classification results, and eventually, the results are not interpretable. Having observed COVID-19 disease subtypes and SARS-CoV-2 variants, heterogeneous populations of all genes are the basic structure of COVID-19 genomics (transcriptional data). As a result, the classical CV method is not applicable in our studies.

Alternatively, given that the fundamental task is to identify critical genes and their joint effects as high-performance genomic biomarkers, we can directly fit the genes identified from the first dataset to several other datasets to test the fitted models and their prediction accuracy. We adopt this approach in this paper.

Additionally, using the existing methods to identify high-performance genes, dozens of genes have been reported in the literature with a lower accuracy than the single-digit number of genes in our new work. If we conclude that the genes identified in this study are overestimated, then we argue that the gene sets with doubled or even tripled numbers of genes should definitely be overestimated and must be useless or not meaningful at all. Therefore, all biological inferences based on those double/tripled numbers of genes can be misleading.

### 3.5. Second Dataset: Five-Gene Classifiers and the Existence of Variants

In this subsection, we test the performance of the five identified genes in the prior section in a second dataset. One significant difference between these two datasets is that the patients in the first study (dataset) were either COVID-19-positive, or had ARIs with other viral infection or ARIs without viral infection, while the patients in the second study (dataset) had NP/OP PCR swab-confirmed SARS-CoV-2 infection or were negative controls. As a result, the genes found to be critical from the first dataset can be thought of as SARS-CoV-2 specific. It turned out that those five genes were also the best subset for the second dataset. Table 7 presents the results from an individual classifier. Data are ln(raw+1) normalized.

We can see that the signs of ATP6V1B2, SERTAD4 and EPSTI1 in CF1 remain the same as their counterparts in Table 1, Table 2, Table 3, Table 4 and Table 5. This table again supports our earlier claim that ATP6V1B2 and IFI27 are in the circle of critical genes associated with SARS-CoV-2. Table 7 also reveals that the information derived using the key genes derived from other datasets can be weak due to weak data quality (e.g., very noisy, no signals). On the other hand, our method can still perform satisfactorily with an overall accuracy of 83.47, sensitivity of 83.49%, and specificity of 83.33%, proving the importance of the identified critical genes and showing the new method’s superiority.

Note that the individual classifier CF1 in the second dataset has a different combination compared with the counterparts in the first dataset. This phenomenon can be explained by the different patient attributes from these two datasets. Next, we computed the correlations among those five genes for each dataset. Table 8 presents pairwise correlations in a matrix form in which the upper triangle is for the first dataset, and the lower triangle is for the second dataset.

Table 8 shows different correlation structures among the five genes, which makes the difference in classifiers between the two datasets reasonable.

## 4. Genomic Differences between NP/OP PCR Swab Samples and Whole Blood Samples

In this section, we use additional twelve datasets to cross-validate the genes identified in Section 3. These datasets include GSE152641 [35], GSE155454 [36], GSE163151 [37], GSE166190 [38], GSE166253 [39], GSE166530 [40], GSE177477 [41], GSE179448 [42], GSE184401 [43], GSE189039 [44,45], GSE190680 [46], and GSE201530 [45,47].

**Figure 3:**
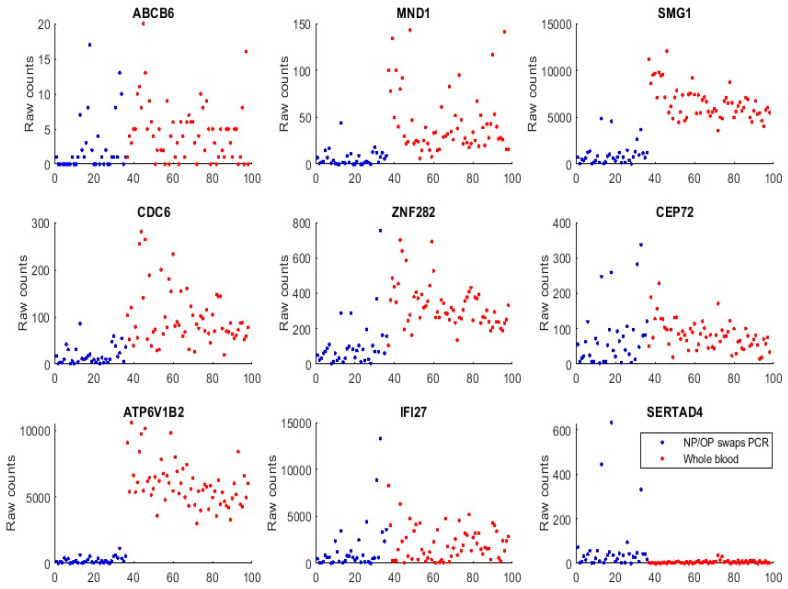
Gene expression raw counts from COVID-19 positives. The red-colored dots represent patients from GSE152641 whole blood samples. The blue dots represent patients from GSE166530 NP/OP PCR swab samples.

We first used GSE152641 and GSE166530 to form a combined dataset to empirically justify that the genes identified in Section 3, and those genes (ABCB6, KIAA1614, MND1, SMG1, RIPK3, CDC6, ZNF282, and CEP72) published in our earlier work [17], are functionally distinct in SARS-CoV-2 and COVID-19. GSE152641 has the overall design of total RNA sequencing from the whole blood of 62 COVID-19 patients and 24 healthy controls, the platform being GPL24676 illumina NovaSeq 6000 (Homo sapiens), and the genome build being GRCh38. GSE166530 has the overall design of nasopharyngeal or oropharyngeal PCR swab samples with 36 COVID-19 positives and 5 negatives. Its platform and genome build are the same as those of GSE152641. We combined the 62 COVID-19 whole-blood-sampled patients from GSE152641 and 36 COVID-19 positive NP/OP swab samples together to form a new dataset. Figure 3 plots expression levels (raw counts) of the new dataset.

We can see that samples from both populations show some similarities in expression level ranges with ABCB6, CEP72, and IFI27, which justifies the feasibility of the graphical comparison since GSE1552641 and GSE166530 have some subtle differences in their data generating processes, though they use the same platform and genomic build.

We can see that ATP6V1B2 shows a completely separable pattern between the two populations. MND1, SMG1, CDC6, and ZNF282 all have higher expression levels in the whole blood than in NP/OP swabs.

We found that SERTAD4’s transcriptomic data in whole blood samples were almost all zeros or very small in Figure 3 (GSE1552641), and other whole blood samples were to be analyzed. This phenomenon tells that SERTAD4 is a phenomenon of symptoms.

Analyzing GSE152641 separately, we obtain the following Table 9:

Comparing Table 9 and our earlier results [17], we can see that the combination of CDC6 and ZNF282 is extended to RIPK3 and KIAA1614, which suggests that CDC6 and ZNF282 can be core genes, and other genes, e.g., CEP72, RIPK3 and KIAA1614, can be substituted.

GSE155454 has an overall design: RNA was extracted from whole blood collected from 27 COVID-19 patients from the Singapore cohort after retrospective matching and 6 healthy controls. Timepoints selected for extraction were during active infection (PCR-positive; median 8 days PIO) and recovered (PCR-negative; median 21 days PIO). The platform was GPL20301 Illumina HiSeq 4000 (Homo sapiens). Table 10 presents our classification results based on the genes identified in Section 3.

We note that this data collection included patients who had recovered from COVID-19, i.e., COVID-19 negative. The coefficient signs of ZNF282 and IFI27 obviously differ from our earlier work [17] and in Table 6. One possible reason is that the recovered patient has different gene expression levels compared with their COVID-19-naïve counterparts, i.e., SARS-CoV-2 infection effects at the genomic level had not completely faded away. Nevertheless, CF1 in Table 10 still leads to a high-performance accuracy of 93.75%.

GSE163151 conducted RNA sequencing (RNA Seq) to analyze nasopharyngeal (NP) swab and whole blood (WB) samples from 333 COVID-19 patients and controls, including patients with other viral and bacterial infections. The platform was GPL24676 Illumina NovaSeq 6000 (Homo sapiens). We took a subset of the data to study the genes identified in Section 3 and in our earlier work. In particular, 138 NP swab samples and 7 whole blood samples were used. Table 11 presents our classification results.

With an accuracy of 95.74%, clearly, we see that COVID-19 NP swab samples and whole blood samples have different gene–gene interactions among those critical genes identified in Section 3 and our earlier work [17]. Therefore, scientists should pay attention to this dissimilarity, which is fundamental to fighting against the COVID-19 pandemic.

GSE166190’s overall design is a transcriptomic analysis of whole blood from SARS-CoV-2-infected participants and their SARS-CoV-2-negative household contacts. In the analysis, the transcriptomic data of an individual were collected in 5-time intervals according to the calculated days POS: interval 1 (0–5), interval 2 (6–14), interval 3 (15–22), interval 4 (23–35), and interval 5 (36–81). The platform was GPL20301 Illumina HiSeq 4000 (Homo sapiens). Table 12 presents our analysis of the data.

In contrast to GSE155454, this study’s time intervals are quite wide. We used six critical genes identified in our earlier work [17] to reach a 77.55% accuracy, which is much lower than our other analysis in the COVID-19 study, though it is already an accepted rate. A possible reason is that in this data, gene–gene interactions from the interval 1 (0–5days) to the follow-up intervals were different, which decreased the sensitivities of our CFi classifiers. However, we obtained 100% specificity with all individuals being tested up to five times. In our supplementary full data table, we found that interval 1 had 100% sensitivity and some of interval 2 had 100% sensitivity. As such, it may be safe to say that the genes in our earlier work [17] worked perfectly.

GSE166253 studied transcriptomic characteristics and impaired immune function of patients who retested positive (RTP) for SARS-CoV-2 RNA. The platform was GPL20795 HiSeq X Ten (Homo sapiens). The data contains 10 retested positive patients, 6 convalescent patients, and 10 healthy controls who were enrolled for analysis of the immunological characteristics of their peripheral blood mononuclear cells. Table 13 reports our fitting results.

The table shows that the gene–gene interactions were different among RTP and convalescent patients. It is interesting to note that we obtained 100% accuracy in this data analysis.

GSE166530 was used in Figure 3 with its COVID-19 positive patients’ NP-swab-sampled gene expression levels. In addition, we conducted a separate classification analysis using the five genes identified in Section 3. Table 14 reports the results.

This table shows different coefficient patterns from Table 7. We note that we only have five healthy individuals in control. Interestingly, if we use the five genes identified in our earlier work [15,17], we can achieve 100% accuracy. This Indian cohort is worth further looking into its gene–gene and subvariant interactions. However, we did not find additional characteristics available to study.

GSE177477 is a Pakistan cohort study. Its overall design is that COVID-19 cases with positive respiratory samples of SARS-CoV-2 and healthy control cases were recruited. Blood transcriptomes were analyzed using Clariom S RNA Microarray, Affymetrix Inc. The platform was GPL23159 [Clariom_S_Human] Affymetrix Clariom S Assay, Human (Includes Pico Assay). We used 11 symptomatic samples and 18 healthy control samples to test our earlier work which identified the genes’ predicting accuracy. We obtained 100% accuracy in this analysis. The results are presented in Table 15.

The coefficient signs of CDC6, ZNF282, and CEP71 are consistent with our earlier work [17]. Again, this study highlights the importance of CDC6 and ZNF282.

GSE179448 conducted RNAseq analysis of human CD4+ regulatory Tregs and Tconvs in COVID-19 patients and healthy donors isolated from peripheral blood. We used 22 hospitalized COVID-19 samples and 15 healthy control samples to test our earlier work which identified the genes’ predicting accuracy. The results are presented in Table 16.

We obtained an 89.19% overall accuracy in this study. One possible reason may be that the platform was GPL18573 Illumina NextSeq 500 (Homo sapiens), compared with GPL24676 Illumina NovaSeq 6000 (Homo sapiens) which led to higher accuracy.

GSE184401 used a platform of GPL24676--Illumina NovaSeq 6000 (Homo sapiens). Its overall design is an RNA-seq analysis in the peripheral blood mononuclear cell isolated shortly from the initial infection. All individuals were COVID-19-confirmed with three types: severe condition with secondary infection, severe condition without secondary infection, and mild infection. We present our results of four genes from our earlier work [17] and three from Section 3 in Table 17.

From this analysis, we see that gene–gene interactions are different after infection with different severe conditions.

GSE189039 has the overall design of RNA-seq being performed on the peripheral blood mononuclear cells (PBMCs) of COVID-19 patients infected by the SARS-CoV-2 Beta variant (Beta) and SARS-CoV-2-naïve vaccinated individuals. The platform was GPL24676 Illumina NovaSeq 6000 (Homo sapiens). Our analysis results are presented in Table 18.

It is interesting to point out that we used only one classifier, CF1, to reach 100% accuracy.

GSE190680 has an overall design of RNA-seq being performed with the peripheral blood mononuclear cells (PBMCs) of COVID-19 patients infected by the SARS-CoV-2 Alpha variant with or without the escape mutation. The platform was GPL24676 Illumina NovaSeq 6000 (Homo sapiens). Note that all patients in this study were COVID-19 patients infected by the SARS-CoV-2 Alpha variant. We used our identified critical genes to test the ability to separate E484K escape mutation. Table 19 presents the results.

With an overall accuracy of 84%, it is safe to say that the three genes ABCB6, CDC6, and CEP72 have the ability to predict E484K escape mutation.

In GSE201523, RNA-seq was performed with peripheral blood mononuclear cells (PBMCs) of COVID-19 patients infected by the SARS-CoV-2 Omicron variant. The platform was GPL24676 Illumina NovaSeq 6000 (Homo sapiens). The following Table 20 is adapted from our work on vaccine study [47].

It is significant to note that the genes identified from blood samples in our earlier work [17] again work for various SARS-CoV-2 variants, including Omicron.

## 5. Discussions

The results presented in this paper are the first to directly associate a few critical genes with SARS-CoV-2 with the best performance (relative to other subsets with the same number of genes). Furthermore, the results signify the genomic difference between NP/OP PCR swab samples and whole blood samples (hospitalized patients), identify single-digit critical genes (ATP6V1B2, IFI27, BTN3A1, SERTAD4, and EPSTI1), which are a transcriptional response to SARS-CoV-2, interpret the functional effects of gene–gene interactions and gene–variant interactions using explicitly mathematical expressions, introduce graphical tools for medical practitioners to understand the genomic signature patterns of the virus, make suggestions on developing more efficient vaccines and antiviral drugs, and finally identify potential genetic clues to other diseases due to COVID-19 infection.

We used a total of fourteen cohort studies (including different platforms, different ethics, different geographical regions, breakthrough infections and Omicron variants) with 1481 samples to justify our results. So far, we have not seen any other research in the literature that had such nearly perfect performance. With such comprehensive studies and conclusive outcomes, it may be safe to say that the identified genes in this paper are representative, and that the gene–gene interaction heterogeneity between SARS-CoV-2 and COVID-19 does exist. Such significant findings can help explore the causal and pathological clues between SARS-CoV-2 infection and the COVID-19 disease and fight against the disease with more targeted genes, vaccines, antiviral drugs, and therapies.

In Zhang [17], a conceptual visualization of the gene–gene relationship was created. At the top of the figure, virus variants were placed. With the new findings of this paper, six signature patterns from Table 3, Table 4 and Table 5 can be used to replace those virus variants, and then a complete dynamic flow can be formed.

As discussed in the introduction, the genes identified in Zhang [17] are hypothesized to link to the root cause of COVID-19, while the genes identified in this study are the key to treating the symptoms. Therefore, based on the findings in this paper, we make the following hypotheses.

**Hypothesis** **1** **(H1).** *The five genes ABCB6, KIAA1614, MND1, SMG1, RIPK3, and their functional effects are the key to curing the root cause [17].*

**Hypothesis** **2** **(H2).** *The five genes ATP6V1B2, IFI27, BTN3A1, SERTAD4, EPSTI1, and their functional effects are the key to treating the symptoms.*

**Hypothesis** **3** **(H3).** *The genes CDC6 (cell division cycle 6) [17] and MND1 are protein essentials for the initiation of RNA replication.*

Hypothesis 1 is based on the mathematical and biological equivalence between the COVID-19 disease and the functional effects of these five genes proved in Zhang [17]. At the moment, testing Hypothesis 2 is more urgent than testing Hypothesis 1, given that variants of SARS-CoV-2 have been emerging. Furthermore, once Hypothesis 2 is tested and confirmed, scientists can test their counterparts in animals, trace the virus origin, and find the intermediate host species of SARS-CoV-2. As to Hypothesis 3, in Zhang (2021) [17], a combination of CDC6 and ZNF282 (Zinc Finger Protein 282) lead to 97.62% accuracy (98% sensitivity, 96.15% specificity), with the following classifier: 1.7615 + 6.8226 × CDC6 − 1.1556 × ZNF282, which suggests that the protein encoded by CDC6 is a protein essential for the initiation of RNA replication. In addition, ZNF282 can be a repressor of COVID-19 RNA replication.

As mentioned in the introduction, ATP6V1B2 was found to impair lysosome acidification and cause dominant deafness-onychodystrophy syndrome [48], while IFI27 was found to discriminate between influenza and bacteria in patients with suspected respiratory infection [25]. There have been new concerns around the COVID-19 disease, e.g., SARS-CoV-2 entering the brain [12], COVID-19 vaccines complicating mammograms [13], memory loss and ‘brain fog’ [14], and SARS-CoV-2 persisting for months after traversing the body [26]. Using the findings from this paper, we may hypothesize that ATP6V1B2 can be a leading factor linking COVID-19 to brain function and ENT problems. As to IFI27, given that COVID-19 is a respiratory tract infection, it makes sense to hypothesize that IFI27 is the infection’s key. EPSTI1 has been found to be related to breast cancer, oral squamous cell carcinoma (OSCC) and lung squamous cell carcinoma (LSCC) [49], which may link COVID-19 to what has been found in the complication of mammograms [13]. Liang et al. (2021) [50] suggested that BTN3A1 may function as a tumor suppressor and may serve as a potential prognostic biomarker in NSCLCs and BRCAs. A confirmed Hypothesis 2 may help further explore whether these genes reported in the literature are truly effective, as suggested in the literature.

Finally, with the proven existence of signature patterns associated with SARS-CoV-2 and COVID-19, variants of the disease will continue to emerge if the problems revealed by the existing signatures are not solved.

## Figures and Tables

**Table 1:** First dataset: Characteristics of the top-performing individual genes together with ATP6V1B2 and IFI27 to form a three-gene classifier.

Classifier	Intercept	ATP6V1B2	IFI27	BTN3A1	SERTAD4	EPSTI1	Accuracy	Sensitivity	Specificity
BTN3A1	−9.818	−8.0116	2.1871	5.2583	0	0	88.46%	83.87%	91.49%
SERTAD4	−4.5269	−1.9712	2.1584	0	−7.803	0	89.32%	86.02%	91.49%
EPSTI1	−7.2904	−7.25	2.6524	0	0	4.1633	89.74%	93.55%	87.23%

**Table 2:** First dataset: Characteristics of RIPK3 together with ATP6V1B2 and IFI27.

Classifier	Intercept	ATP6V1B2	IFI27	RIPK3	Accuracy	Sensitivity	Specificity
RIPK3	−1.2487	−5.7586	1.3916	9.902	87.2%	76.3%	94.3%

**Table 3:** First dataset: Characteristics of the top-performing five-gene classifier. CF1 and CF2 are the first and second individual classifiers for data of COVID-19 patients vs. other viral ARIs and non-viral infection patients.

Classifier	Intercept	ATP6V1B2	IFI27	BTN3A1	SERTAD4	EPSTI1	Accuracy	Sensitivity	Specificity
CF1	9.193	−1.8935	1.5774	0	−4.3303	0	87.61%	81.72%	91.49%
CF2	−7.2786	−5.2993	0	3.2572	0	2.34	86.32%	76.34%	92.91%
max{CF1, CF2}							91.88%	94.62%	90.07%

**Table 4:** First dataset: Characteristics of the top-performing five-gene classifier. CF1 and CF2 are the first and second individual classifiers for data of COVID-19 patients vs. other viral infection ARIs, but not non-viral infection patients.

Classifier	Intercept	ATP6V1B2	IFI27	BTN3A1	SERTAD4	EPSTI1	Accuracy	Sensitivity	Specificity
CF1	−2.052	0	3.9086	2.5578	0	−9.6586	70.15%	62.37%	87.8%
CF2	5.5979	−7.4352	0	0	8.3704	4.4936	76.12%	74.19%	80.49%
max{CF1, CF2}							91.04%	97.85%	75.61%

**Table 5:** First dataset: Characteristics of the top-performing five-gene classifier. CF1 and CF2 are the first and second individual classifiers for data of COVID-19 patients vs. non-viral infection ARI patients.

Classifier	Intercept	ATP6V1B2	IFI27	BTN3A1	SERTAD4	EPSTI1	Accuracy	Sensitivity	Specificity
CF1	−2.2381	−7.9733	0	4.5448	0	4.7567	90.16%	81.72%	98%
CF2	−2.1003	−4.8036	4.0849	0	−9.9738	0	90.16%	82.8%	97%
max{CF1, CF2}							96.37%	95.70%	97%

**Table 6:** First dataset: Expression values of the five critical genes, competing classifier factors and predicted probabilities.

#ID	Status	ATP6V1B2	IFI27	BTN3A1	SERTAD4	EPSTI1	CF1	CF2	CFmax	P_1_	P_2_	Pmax
e-202	0	277	604	104	158	138	−246.7	−813.52	−246.7	0.08	0.00	0.08
e-080	0	866	103	82	76	94	−1797.2	−4109.42	−1797.2	0.00	0.00	0.00
e-287	0	3127	717	271	233	151	−5789.8	−15342.2	−5789.8	0.00	0.00	0.00
e-753	1	1053	2029	766	214	819	289.2	−1176.0	289.2	0.95	0.00	0.95
e-751	1	253	1423	266	114	369	1281.1	381.87	1281.1	1.00	0.98	1.00
e-520	0	617	344	120	11	559	−664.1	−1578.0	−664.1	0.00	0.00	0.00
e-505	0	721	240	298	10	500	−1020.8	−1687.4	−1020.8	0.00	0.00	0.00
i-083	0	191	320	119	72	71	−159.5	−465.7	−159.5	0.17	0.01	0.17
e-764	0	1667	202	76	3	1232	−2841.6	−5710.8	−2841.6	0.00	0.00	0.00
e-451	0	1880	24	98	2	27	−3521.4	−9587.6	−3521.4	0.00	0.00	0.00
e-285	0	794	826	530	392	300	−1888.8	−1786.6	−1786.6	0.00	0.00	0.00
e-254	0	512	253	195	388	69	−2241.4	−1923.9	−1923.9	0.00	0.00	0.00
e-726	1	398	1395	362	96	567	1040.3	389.5	1040.3	1.00	0.98	1.00

**Table 7:** Second dataset: Characteristics of the top-performing five-gene classifier. CF1 stands for the first individual classifier for COVID-19-positive vs. COVID-19-negative data.

Classifier	Intercept	ATP6V1B2	SERTAD4	EPSTI1	Accuracy	Sensitivity	Specificity
CF1	−10.9845	−3.2959	−0.4205	7.6279	83.47%	83.49%	83.33%

**Table 8:** Pairwise correlation coefficients: The upper triangle is for the first dataset, and the lower triangle is for the second dataset.

	ATP6V1B2	IFI27	BTN3A1	SERTAD4	EPSTI1
ATP6V1B2	1	0.208	0.5416	0.051	0.5415
IFI27	0.4031	1	0.5463	0.3084	0.5616
BTN3A1	0.69	0.3823	1	0.25	0.7527
SERTAD4	0.3417	0.3302	0.2663	1	0.0079
EPSTI1	0.6531	0.3366	0.6562	0.1791	1

**Table 9:** GSE152641: Characteristics of the top-performing four-gene classifier. CF1 and CF2 are the first and second individual classifiers for data of COVID-19 patients vs. healthy controls.

Classifier	Intercept	KIAA1614	RIPK3	CDC6	ZNF282	Accuracy	Sensitivity	Specificity
CF1	−5.7093		8.4656	5.8485	−9.3695	80.23%	72.58%	100%
CF2	−6.8734	5.9693	−2.9708	8.6925		77.91%	69.35%	100%
Max{CF1,CF2}						98.84%	98.39%	100%

**Table 10:** GSE155454: Characteristics of the top-performing three-gene classifier CF1 for data of COVID-19 positives vs. negatives and healthy controls.

Classifier	Intercept	RIPK3	ZNF282	IFI27	Accuracy	Sensitivity	Specificity
CF1	−7.9946	−7.1725	6.9103	5.7519	93.75%	89.66%	84.62%

**Table 11:** GSE163151: Characteristics of the top-performing three-gene classifier CF1 for data of whole blood vs. NP/OP swabs.

Classifier	Intercept	ABCB6	KIAA1614	MND1	Accuracy	Sensitivity	Specificity
CF1	−12.337	−0.416	0.3737	1.4604	95.74%	100%	95.52%

**Table 12:** GSE166190: Characteristics of the top-performing six-gene classifier. CF1, CF2, CF3 are the first, second and third individual classifiers for data of COVID-19 patients vs. healthy controls. The data were natural logarithm-transformed as ln(KIAA1614/10+1), ln(MND1+1), ln(RIPK3/10+1), ln(SMG1/100+1), ln(ZNF282/10+1), ln(CEP72+1).

Classifier	Intercept	KIAA1614	MND1	RIPK3	SMG1	ZNF282	CEP72	Accuracy	Sensitivity	Specificity
CF1	25.7352			11.4885	−16.3554	−1.6889		31.63%	19.28%	100%
CF2	8.5694	−9.6995	4.0413				2.342	62.24%	55.42%	100%
CF3	−12.6727	−5.3787		11.2971			−3.1795	27.55%	14.46%	100%
CFmax								77.55%	73.49%	100%

**Table 13:** GSE166253: Characteristics of the top-performing four-gene classifier. CF1 and CF2 are the first and second individual classifiers for data of COVID-19 patients vs. healthy controls.

Classifier	Intercept	MND1	RIPK3	SMG1	CDC6	Accuracy	Sensitivity	Specificity
CF1	7.862	3.0801	−0.3897		−2.3531	92.31%	87.5%	100%
CF2	4.2178		−0.5365	0.1789	−0.1874	96.15%	93.75%	100%
Max{CF1,CF2}						100%	100%	100%

**Table 14:** GSE166530: Characteristics of the top-performing five-gene classifier. CF1 and CF2 are the first and second individual classifiers for data of COVID-19 patients vs. healthy controls.

Classifier	Intercept	ATP6V1B2	IFI27	BTN3A1	SERTAD4	EPSTI1	Accuracy	Sensitivity	Specificity
CF1	−11.1266	8.5087	−1.4154			−7.6515	31.71%	22.22%	100%
CF2	6.8238	0.4763			−1.9013	0.2038	86.11%	86.11%	100%
max{CF1, CF2}							95.12%	94.44%	100%

**Table 15:** GSE177477: Characteristics of the top-performing four-gene classifier. CF1 and CF2 are the first and second individual classifiers for data of COVID-19 patients vs. healthy controls.

Classifier	Intercept	SMG1	CDC6	ZNF282	CEP72	Accuracy	Sensitivity	Specificity
CF1	0.6104	2.3501	1.6222		−8.9165	79.31%	45.45%	100%
CF2	−2.0531	−0.5364	13.4123	−10.0557		93.10%	81.82%	100%
Max{CF1,CF2}						100%	100%	100%

**Table 16:** GSE179448: Characteristics of the top-performing five-gene classifier. CF1 and CF2 are the first and second individual classifiers for data of COVID-19 hospitalized patients vs. healthy controls.

Classifier	Intercept	KIAA1614	MND1	RIPK3	CDC6	CEP72	Accuracy	Sensitivity	Specificity
CF1	4.328		−1.5254		8.7869	−4.5027	81.08%	72.72%	93.33%
CF2	10.0917	7.9273		−4.3736		6.7933	48.65%	13.64%	100%
max{CF1, CF2}							89.19%	86.36%	93.33%

**Table 17:** GSE184401: Characteristics of the top-performing seven-gene classifier. CF1, CF2, CF3 are the first, second and third individual classifiers for data of severe COVID-19 condition vs. mild infection.

Classifier	Intercept	KIAA1614	CDC6	ZNF282	CEP72	ATP6V1B2	IFI27	BTN3A1	Accuracy	Sensitivity	Specificity
CF1	−1.4488		6.9615		−2.125		−3.828		76.74%	59.09%	95.24%
CF2	10.8726	9.3158			−4.309		−0.19		69.77%	40.91%	100%
CF3	5.1235			6.758		2.5267		−4.0934	88.37%	77.27%	100%
CFmax									95.35%	95.45%	95.24%

**Table 18:** GSE189039: Characteristics of the top-performing three-gene classifier CF1 for data of COVID-19 vs. healthy control.

Classifier	Intercept	ABCB6	MND1	CEP72	Accuracy	Sensitivity	Specificity
CF1	4.742	−0.001	0.0402	−0.072	100%	100%	100%

**Table 19:** GSE190680: Characteristics of the top-performing three-gene classifier CF1 for data of Alpha-E484K vs. Alpha.

Classifier	Intercept	ABCB6	CDC6	CEP72	Accuracy	Sensitivity	Specificity
CF1	9.8031	−2.1852	1.4385	3.1508	84%	76.67%	87.14%

**Table 20:** Performance of individual classifiers and combined max-competing classifiers using blood-sampled data GSE201530 to classify the COVID-19 infected and healthy controls into their respective groups. The meaning of CF-i is the same as those in Table 1. Raw stands for raw counts.

Classifiers	Intercept	ABCB6	MND1	RIPK3	SMG1	CDC6	ZNF282	CEP72	Accuracy	Sensitivity	Specificity
CF1(Raw)	−1.6909				0.0001	2.0352	−0.6842		50.91%	42.55%	100%
CF2(Raw)	−7.5469	−0.9264	5.8238					1.9166	80%	76.60%	100%
CF3(Raw)	1.466	0.4688	−1.4305	−0.0862					20%	6.38%	100%
CF4(Raw)	3.0641	−0.8549			0.0001		0.6613		70.91%	65.96%	100%
CFmax									100%	100%	100%

## Data Availability

The datasets are publicly available. The data links are stated in the Section 3.

## Supplementary Materials

Real data and computer outputs are in a supplementary file available online https://pages.stat.wisc.edu/~zjz/BHDataCode.zip. In addition, a MATLAB^®^ demo code for solving a final dataset example in Equation (4) (λ_2_ = 0) is also available.

## Limitation Statements

Although we have identified functional effects by gene–gene interactions and gene–subtype (variants) interactions of the five genes, we have not identified how genes interact with each other and their causal directions. We are working in this direction. Finally, our results are in the field of computational biology/medicine, and they are not lab-confirmed.
